# Paediatric bipolar disorder: an age-old problem

**DOI:** 10.1186/s40345-023-00309-4

**Published:** 2023-08-26

**Authors:** Gin S. Malhi, Maedeh Jadidi, Erica Bell

**Affiliations:** 1 Academic Department of Psychiatry, Northern Clinical School, Faculty of Medicine and Health, Kolling Institute, The University of Sydney, Sydney, NSW Australia; 2 CADE Clinic Level 3 and Mood-T, Main Hospital Building, Royal North Shore Hospital, Northern Sydney Local Health District, St. Leonards, NSW 2065 Australia; 3 Department of Psychiatry, University of Oxford, Oxford, UK

## Dear Editor,

A recent re-examination of Paediatric Bipolar Disorder (PBD), by Peter Parry and colleagues in this journal (Parry et al. 2021), reveals once again that the diagnosis of a bipolar illness remains a challenge, especially in young people, where the term PBD is used by some to describe the occurrence of bipolar symptoms in individuals less than 18 years old. We agree by and large with the concerns raised by Parry and colleagues, as narrowing this descriptor further, the term prepubertal bipolar disorder (PPBD) is then used to describe those experiencing what are thought to be bipolar symptoms prior to puberty (Malhi and Bell 2020).

We have previously argued that perhaps bipolar disorder^1^ should not be diagnosed prior to puberty and that the term PBD should be revised at the very least, if not retired altogether (Malhi et al. 2020). Therefore, in this brief correspondence, we do not reiterate our classificatory arguments or the research and clinical rationale for such a change; instead, we turn our attention to the term itself, and consider why it is inherently misleading. We then examine the diagnostic challenge of eliciting and detecting phenomenology in youth before proposing a possible solution by designating this as adolescent bipolar disorder (ABD).

## What’s in a name?^2^

The diagnosis of paediatric bipolar disorder remains in active use, and even critics of the descriptor (Parry et al. 2021) are forced to use the term as there is no adequate alternative. We feel that this parlous state of affairs is hindering research and preventing us from achieving a more meaningful understanding of the origins of the illness. A significant problem is the name itself.

This is because firstly, the term ‘paediatric’ implies the role of paediatricians. Specifically, it suggests that the illness is largely encountered in paediatrics, and as such, it is the remit of this medical subspecialty. However, bipolar disorder *is* clearly a mental illness that affects adults and is diagnosed as a psychiatric disorder across the lifespan. Thus, its designation should reflect this. Further, the diagnosis should only be assigned conclusively by a psychiatrist after being assessed by those with the appropriate psychiatric and psychological expertise. At present, the prefix ‘paediatric’ lacks these important connotations, and instead, it arguably diminishes the significance of psychiatry’s decisive role in clinically delineating bipolar disorder.

Secondly, the word paediatric^3^ places all individuals under the age of eighteen years in a single group. In other words, everyone who is not yet an adult is regarded as one diagnostic entity. This is a significant problem because these initial eighteen years of life contain discernible developmental milestones that separate phases of growth into distinct periods, such as childhood, puberty, and adolescence. Each of these developmental phases has significant differences that impact detection and diagnosis. For instance, by definition, children are still learning how to envisage their emotions and interpret them cognitively and, at the same time, are still acquiring the vocabulary to describe them meaningfully. Thus, an individual’s ability to understand themselves and convey their experiences varies considerably by age throughout these early developmental phases and trying to capture these changes using measures that are derived from adult bipolar disorder DSM-5 or ICD-11 checklists seems to be a strategic error.

## Diagnostic challenge

Diagnosing bipolar disorder in prepubertal individuals is based on the hypothesis that elements of the illness are likely to be present from an early age. This seems reasonable however, most symptoms of mania and the episodic pattern of the illness cannot be assumed to be evident in childhood. For example, heightened libido, sexual promiscuity, risk-taking, overspending, grandiosity, and diminished insight are impossible to anchor in the behaviour of prepubertal children, as is the establishment of a timeline. Further, other symptoms of mania in adults are less remarkable when they occur in children and may even be regarded as age appropriate. For instance, the firm belief of being a superhero with special powers is likely to be of concern in an 18-year-old but is regarded as commonplace in an 8-year-old. In other words, the belief is age specific. In contrast, irritability appears to be a complex trans-diagnostic phenomenon that is challenging to characterise at any age, despite its ubiquity (Bell et al. 2021).

In practice, one of the reasons the label PBD remains in clinical use is because it serves as a descriptor of childhood bipolar symptoms that adults with bipolar disorder recall upon inquiry. Thus, the diagnosis is made retrospectively, even though such symptoms are subject to recall and confirmation bias. Further, it is important to note that even manic-like symptoms in youth only hold predictive value in the context of a confirmed family history (Duffy et al. 2020), and that in most instances, very young children presenting with symptoms akin to irritability, and explosive temper and comorbid attention problems, do not eventuate in bipolar disorder phenomenology in later years.

## Puberty and cognitive and emotional development

Puberty ends childhood. It is the time when individuals undergo physiological changes to achieve sexual maturation and fertility. Its onset varies between individuals and is initiated physiologically by reactivation of the hypothalamic-pituitary–gonadal axis, which is active in the embryonic and early postnatal stages and inhibited during childhood (Abreu and Kaiser 2016). The sex hormones that become active during puberty also impact brain development and influence emotion regulation and the processing of social information (Ladouceur 2012). Other important psychological changes that occur during adolescence are the development of abstract thinking, the ability to mentalise (theory of mind) and introspect, the emergence of personal and sexual identity, and a system of values and coping skills to overcome problems and crises (Remschmidt 1994). Thus, puberty is an essential and important phase of development and growth and its onset may be objectively described using Tanner staging^4^ (Emmanuel and Bokor 2022).

## Alternatives to paediatric bipolar disorder

Having critiqued the term ‘paediatric’ in PBD, and argued against assigning this diagnosis prior to puberty, we are left with having to furnish an alternative.

### Juvenile bipolar disorder

An alternative diagnosis that is occasionally used instead of PBD is Juvenile Bipolar Disorder. The term ‘Juvenile’ is widely used in the US justice system even though it is problematic because it is poorly defined, and the minimum age of juvenile adjudication varies tremendously across the US. Therefore the juvenile justice system is trying to both raise the minimum age of prosecution and make this consistent across all jurisdictions (NGA 2021). Thus, given the variability in the meaning of ‘juvenile’, perhaps it is *not* the ideal descriptor for young people with bipolar disorder, especially as it may also incorrectly insinuate some form of criminality. This is why instead; we propose that post-pubertal individuals with apparent symptoms of bipolar illness should be referred to as having Adolescent Bipolar Disorder (ABD).

### Adolescent bipolar disorder

**Fig. 1:**
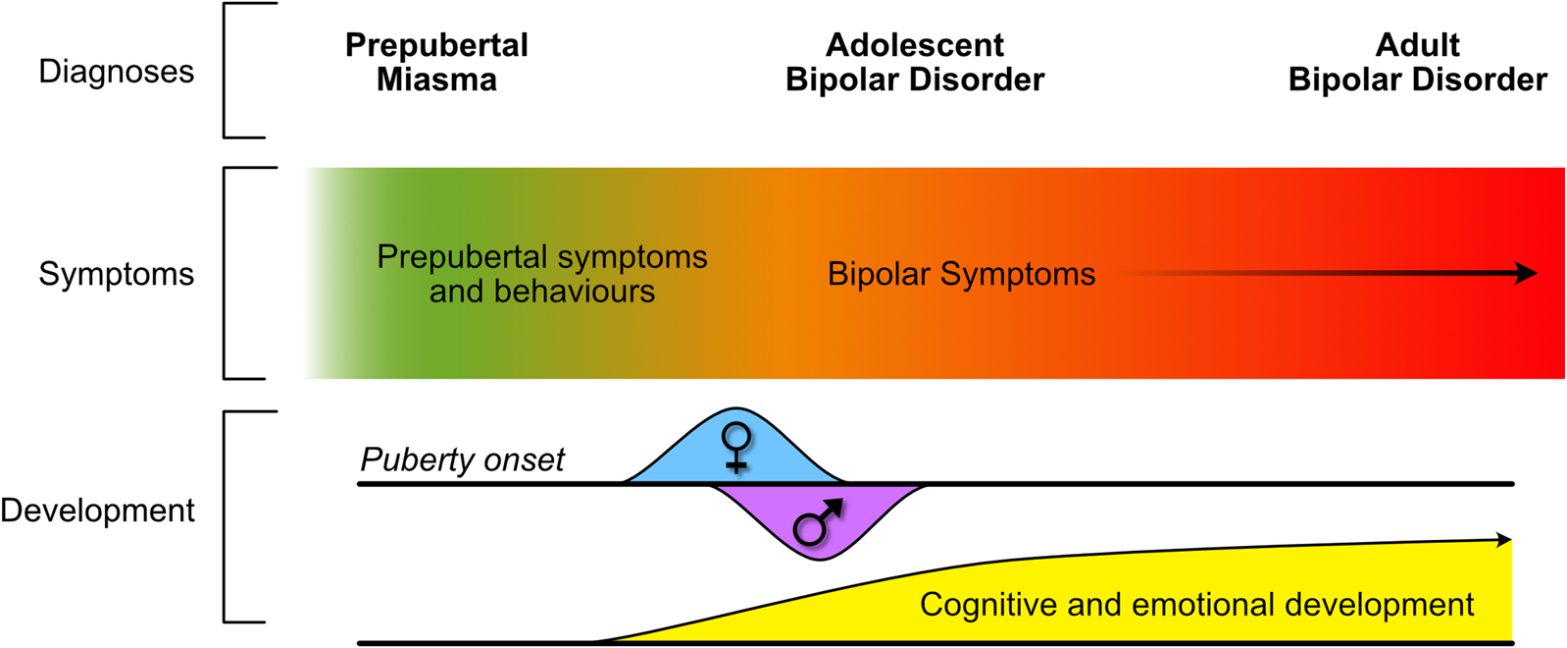
Developmentally informed diagnostic labels for bipolar disorder. This schematic of physiological and psychosocial development provides a timeline against which we have illustrated various descriptions for bipolar diagnoses. Presently, PBD spans all developmental phases up to adulthood. Instead, we propose that this term (PBD) be supplanted with Adolescent Bipolar Disorder, which commences after childhood, typically coinciding with the onset of puberty, and extends to adulthood. In addition, we propose a new term “Prepubertal Miasma”. This refers to symptoms in childhood that *may* be precursors to bipolar disorder in adolescence and adulthood. For example, symptoms such as irritability may eventuate in a diagnosis of bipolar disorder but can instead follow a trajectory that eventuates in other psychiatric conditions such as attention-deficit hyperactivity disorder (ADHD), or may resolve spontaneously as the individual matures. The term ‘miasma’ reflects the foreboding nature of these symptoms as they create an atmosphere of potential bipolar phenomenology rather than symptoms that reflect a discrete illness. This term holds equivalence to terms such as pre-bipolar risk states, indicative of bipolar antecedents. The diffuse borders of puberty commencement reflect individual variability of its boundaries. For example, males usually start puberty later than females (typical age for males 10–12 years and for females 9–11 years). The diffuse nature of the ‘boundary’ between prepubertal symptoms and those indicative of bipolar disorder reflects the potential permeability of the symptoms and behaviours that constitute the prepubertal miasma

We prefer ABD, as it positions the diagnosis firmly after childhood has ended, typically signified by the onset of puberty,^5^ by which time individuals are more likely to reliably recognise their emotions and interpret them meaningfully (see Fig. 1). This is because, compared to children, adolescents have a greater vocabulary to accurately describe their emotions and inevitably have more life experience to contextualise their feelings. Further, they are more likely to comprehend society’s expectations regarding normal social, behavioural, and learning functions, and this enables clinicians to elicit bipolar symptoms with greater confidence and correlate them with collateral history.

Prior to puberty, symptoms thought to perhaps indicate future bipolar disorder may be referred to as a *miasma* (see Fig. 1). This *miasma* may contain symptoms that potentially permeate into and beyond puberty and adolescence, but when monitoring these symptoms prospectively and prior to puberty, they must be viewed critically.

## Conclusion

In summary, we argue for the replacement of PBD with developmentally informed diagnostic categories that are more likely to facilitate research and improve clinical practice. The term PBD may still have some use as a shorthand to capture the symptoms of bipolar disorder that come to mind when retrospectively recalling experiences that have taken place prior to adulthood—especially when there is doubt as to when these precisely occurred. But to diagnose and prognosticate bipolar disorder in young people, we need diagnoses that are meaningful and represent more homogeneous groups of patients. Therefore, we propose that the term adolescent be used to describe bipolar symptoms that occur once puberty has commenced and that symptoms occurring prior to this time that are thought to be indicative of future bipolar disorder be referred to as a miasma. This refined terminology should be adopted in clinical practice and be the focus of future research.

## Data Availability

Not applicable.

## Footnotes

1 This refers to adult bipolar disorder as diagnosed in DMS-5 and ICD-11.

2 *“What's in a name? That which we call a rose by any other name would smell just as sweet.”* Romeo and Juliet by William Shakespeare—implies that the naming of things is irrelevant.

3 Paediatrics: ‘pediatrics’ (USA), ‘pädiatrik’ (German) or ‘pédiatrie’ (French), originating from classical Greek ‘paedo’ meaning a child and ‘iatric’ meaning a physician is the speciality of medicine that takes care of sick and injured children.

4 The Tanner scores assigned by a clinician consist of 3 scores for boys and 3 for girls, describing genitals, testicles, and pubic hair in boys and breasts, pubic hair, and menarche in girls.

5 As defined by the World Health Organization (WHO), wherein puberty from onset to completion can span from 10 – 19 years.
